# Comparison of different quantitative evaluation protocols for peri-device leak detection using cardiac computed tomography angiography after left atrial appendage closure

**DOI:** 10.1007/s10554-022-02748-z

**Published:** 2022-11-03

**Authors:** Shiqi Li, Jing Dong, Jie Luo, Gaofeng Wang, Dujiang Xie, Ling Zhou

**Affiliations:** 1 Department of Cardiology, Nanjing First Hospital, Nanjing Medical University, Nanjing, 210006 China; 2 Department of Echocardiography, Nanjing First Hospital, Nanjing Medical University, Nanjing, 210006 China

**Keywords:** Left atrial appendage closure, Cardiac computed tomography angiography, Transesophageal echocardiography, Peri-device leak, Hounsfield units, Diagnostic performance

## Abstract

This study seeks to propose and compare different quantitative evaluation methods for identifying patients with peri-device leak (PDL) using cardiac computed tomography angiography (CCTA). Patients who had undergone left atrial appendage (LAA) closure and both transesophageal echocardiography (TEE) and CCTA were enrolled. Hounsfield units (HU) were measured in the proximal and distal regions of the left atrial appendage (p-LAA, d-LAA) on the CCTA, and the average of the two was determined (a-LAA). The relative HU ratios of the LAA to the center of the left atrium (LA) were calculated (p-LAA/c-LA, d-LAA/c-LA, a-LAA/c-LA). The area under the curve (AUC) for the LAA HU and the LAA/LA HU ratio were analyzed and compared. Fifty-one patients were included in this study. Pairwise comparisons showed a statistically significant difference (*p* = 0.029) in diagnostic performance between the d-LAA (AUC = 0.868) and a-LAA (AUC = 0.972). There were no significant differences between the a-LAA and p-LAA (*p* = 0.549) or between the d-LAA and p-LAA (*p* = 0.053). At the optimal cutoff for a-LAA of 115.5 HU, the sensitivity was 100%, the specificity was 88%. At the optimal cutoff for p-LAA of 109 HU, the sensitivity was 100%, the specificity was 84%. The LAA/LA HU ratio did not exhibit better diagnostic performance than HU attenuation in the LAA (*p* > 0.05). The a-LAA > 115.5 is useful in identifying PDL. Due to its convenience and intuitiveness, p-LAA > 109.0 can also be used as an alternative protocol for a-LAA.

## Introduction

Approximately 90% of left atrial (LA) thrombus in patients with non-valvular atrial fibrillation (NVAF) originates in the left atrial appendage (LAA) [1]. Left atrial appendage closure (LAAC) is effective in preventing stroke events in patients who are at high risk for thrombosis and cannot tolerate anticoagulation [2, 3]. Peri-device leak (PDL) resulting from incomplete LAA closure is a common complication after interventional therapies targeting the LAA and can be observed during the procedure or follow-up imaging [4]. Theoretically, the turbulence and stagnation of blood flow caused by PDL may lead to thrombus formation surrounding the device or in the left atrial appendage [5, 6]. Studies have shown that PDL is associated with increased rates of ischemic complications, including device-related thrombus (DRT), ischemic stroke, and systemic embolism [7–9].

Adequate imaging modalities guiding preoperative planning and postoperative follow-ups are essential for successful LAA closure. The gold standard for postoperative imaging is transesophageal echocardiography (TEE) [10], which can guide subsequent anticoagulation or antithrombotic therapy. However, in addition to being operator-dependent, this technique is invasive and may lead to esophageal or gastric injury [11]. Therefore, cardiac computed tomography angiography (CCTA) is becoming more widely used for pre- and postoperative evaluation of LAA closure due to its relatively non-invasive nature and high spatial resolution [12]. Measurements of the linear attenuation coefficient (Hounsfield units, HU) in the LAA and HU ratio of the LAA to the left atrium (LA) have been used to evaluate the LAA patency [13]. The criteria for the position of the region of interest (ROI) are unclear. Although most studies follow the principle that the ROI should be selected in the LAA distal to the implanted device to avoid metallic instrumentation artifacts [14, 15], the distance from the device is undefined. As a result, each study may select ROI in different regions of the LAA, such as near the orifice [16] or the middle region of the LAA [17].

We refined the measurement protocols and compared their diagnostic performance. Our purpose was to select an appropriate quantitative evaluation protocol to assess PDL with CCTA using TEE as a reference standard.

## Methods

### Study design and population

This study included consecutive patients with NVAF who underwent LAAC with the WATCHMAN 2.5 (Boston Scientific) or LAmbre (Lifetech Scientific Corp) at Nanjing First Hospital from March 2021 to December 2021. Patients who were at high risk of thromboembolism (CHA2DS2-VASc score ≥ 2) but had contraindications to systemic anticoagulation or were at high bleeding risk (HAS-BLED score > 3) were the most accepted indications for LAAC [18]. Follow-up visits were scheduled for three months post LAAC, including both CCTA and TEE examinations. All patients underwent pre-procedural imaging via TEE or CCTA to exclude the LAA thrombus and evaluate anatomical characteristics. The CCTA excluded patients whose glomerular filtration rate (eGFR) was less than 30 ml/min/1.73 m^2^. Routine transthoracic echocardiography (TTE) was performed to evaluate cardiac function before and after LAAC. All patients provided informed consent. The study was approved by the local ethics committee.

### LAA occlusion procedure

Procedures were performed via TEE guidance under either local or general anesthesia. The orifice diameter and depth of the LAA were measured from the angles of 0°, 45°, 90°, and 135°. After transseptal punctures, intravenous heparin was administered to reach a target ACT of 250–350 s until the procedure was complete. Normal saline load was used to ensure a left atrial pressure greater than 12 mmHg. Angiography was performed using right anterior oblique views coupled with cranial (CRA) and caudal (CAU) angulations. Device sizing was based on angiography, intraoperative TEE, and preoperative CCTA or TEE. The implantation of the WATCHMAN device met the PASS criteria (P: position; A: anchor; S: size; S: seal) before release. For LAmbre, successful sealing met the COST criteria (C: umbrella deployed beyond the circumflex artery; O: umbrella fully open; S: optimal peri-device sealing; T: device stability confirmed by the tug test). A TTE examination was performed at discharge. Patients were treated with anticoagulants (rivaroxaban/dabigatran or warfarin) for three months after the procedure. After the completion of TEE and CCTA, three months of dual antiplatelet therapy (clopidogrel and aspirin) were followed. Mono-antiplatelet therapy (clopidogrel or aspirin) was either not used or used for life, depending on the patient's six-month reexamination results and evaluation. If DRT was discovered during the follow-up, anticoagulation was to be increased for three months before the TEE was re-evaluated.

### Follow-up TEE

TEE was performed using the GE Vivid E95 ultrasound system with a 6VT-D esophageal probe. All patients fasted for a minimum of 6 h before the examination. After local pharyngeal anesthesia with lidocaine mucilage, a transesophageal probe was placed in the middle of the esophagus to record dynamic two-dimensional grayscale images of the LAA at the 0°, 45°, 90°, and 135° planes. PDL was assessed as blood flow communication between the LA and LAA. The LAA was scanned from 0° to 135° to observe the presence or absence of PDL. DRT was defined as a well-circumscribed echo-reflective mass with independent mobility across multiple imaging planes [19]. At least three cardiac cycles’ worth of data was captured, post-processed, and analyzed online. All examinations were performed by an experienced sonographer blinded to clinical data.

### Follow-up CCTA and LAA HU measurement

All examinations were performed using the second generation dual-source CT, Siemens SOMATOM Definition Flash system (Siemens Healthcare, Forchheim, Germany). Scan parameters were retrospectively electrocardiogram (ECG)-gated acquisition, tube voltage of 120 kV, and tube current ranging from 300 to 350mAs. 70–100 ml of non-ionic contrast medium (Iopromide, iodine concentration 370 mg/ml, Bayer Schering Pharma, Germany) was delivered via the patient's peripheral vein at a flow rate of 5.0 ml/sec. 60 ml of 0.9% sodium chloride was injected at the same rate after the contrast agent had been injected. After the ascending aorta had reached the trigger threshold of 100HU, the scan began automatically after a delay of 10 s. Image data sets were reconstructed with a thickness of 0.75 mm, increment of 0.5 mm, and a medium smooth convolutional kernel (B26f). CCTA measurements were obtained using the picture archiving and communication system (PACS) workstation.

**Fig. 1:**
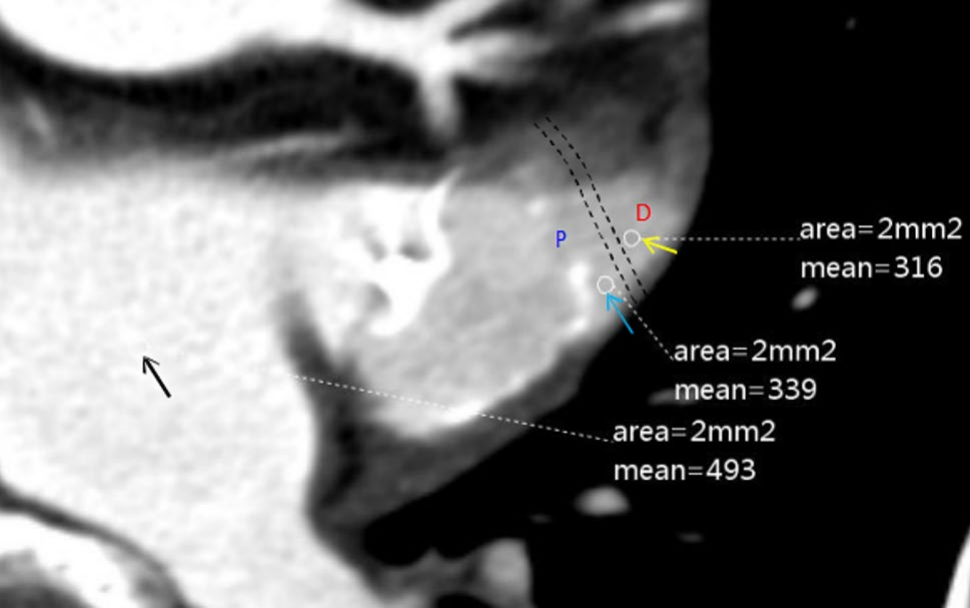
The measuring of Hounsfield units with the region of interest of 2 mm.^2^ on a contrast-enhanced CT image of contrast patency LAA: the distal area (D) of 316 HU (yellow arrow); the proximal area (P) of 339 HU (blue arrow); the center of the left atrium of 493 HU (black arrow)

The study used three protocols of HU measurement in the LAA (Fig. 1). As the ROI was set as a circle with a diameter of 1.6 mm, we used 3.2 and 4.8 mm lines around the device to divide the LAA into three areas. To reduce metallic instrumentation artifacts to the greatest extent possible, we defined the region beyond 4.8 mm surrounding the device as the distal region; the HU measured in this region was denoted as d-LAA. Due to the low left atrial appendage flow velocity [20] and the complex anatomy of the LAA in some patients, the PDL may appear unevenly distributed and limited to the vicinity of the device on the CCTA images. Therefore, we defined the region close to the orifice within 3.2 mm surrounding the device as the proximal area; the HU measured in this region was denoted as p-LAA. Considering that both situations may impact the PDL evaluation, a third measurement method was introduced, taking the average of the HU measured in p-LAA and d-LAA, namely a-LAA. The HU ratios of LAA HU value from the three measurement protocols to the center of the left atrium were calculated. The circular ROI with an area of 2 mm^2^ was placed in the LA, proximal, and distal regions of the LAA. The HU was measured a minimum of three times and the average of the measurements was taken.

## Statistical analysis

All statistical analysis was performed using SPSS 26.0 (SPSS Inc., Chicago, IL, USA) and MedCalc 20.0 (MedCalc Software, Ostend, Belgium). Continuous variables are presented as mean ± standard deviation or median (interquartile range, IQR). Categorical variables are expressed as frequencies and percentages. Normal distribution was tested using the Shapiro–Wilk test. The chi-square test and Fisher’s exact test were used to compare categorical variables. The Student’s t-test and Mann–Whitney U-test were used to compare continuous variables. The area under the curve (AUC) and the optimal cut-off for the LAA HU and the LAA/LA HU ratio were analyzed using the receiver-operating characteristic (ROC) curve. With TEE as the gold standard, positive predictive value (PPV), negative predictive value (NPV), sensitivity, and specificity were calculated. The AUC was calculated and compared according to the DeLong method. A two-sided *p*-value < 0.05 was considered statistically significant.

## Result

During the study period, a total of 55 patients underwent LAAC. With the exception of four patients who could not tolerate the insertion of the TEE probe, all patients underwent CCTA and TEE examinations. Therefore, 51 patients were enrolled in the study. The baseline characteristics are summarized in Table 1. The mean age of the patients was 64.8 ± 7.4 years, and the CHA2DS2-VASc and HAS-BLED scores were 2.9 ± 1.4 and 2.1 ± 0.8, respectively. 21(41.2%) patients had an ischemic stroke or transient ischemic attack (TIA). WATCHMAN was utilized in 37 (72.6%) patients, while LAmbre was utilized in 14 (27.4%). The procedure was successful in all patients. In 22 (43.1%) patients, the LAAC was combined with atrial septal defect (ASD, 6/22), patent foramen ovale (PFO, 5/22), or cryoballoon ablation (13/22) in a one-stop procedure. 9.1% (2/22) of patients underwent the “LAAC + PFO + cryoballoon ablation” combined procedure. No major perioperative adverse events, such as cardiac tamponade, air embolism or thromboembolism, device dislocation, death, or major hemorrhage, were observed during the entire follow-up period. 9.8% (5/51) of patients exhibited minor bleeding complications, including two cases of melena, two cases of bleeding gums, and one case of conjunctival bleeding.

**Table 1:** Baseline clinical characteristics of 51 patients

Patient characteristics	Total (*N* = 51)
Sex, female	23 (45.1%)
Age, years	64.8 ± 7.4
Height, cm	166.4 ± 8.4
Weight, kg	71.0 ± 12.7
Body-mass index, kg / m^2^	25.6 ± 3.6
Persistent or permanent AF	33 (64.7%)
Coronary heart disease	10 (19.6%)
Diabetes mellitus	15 (29.4%)
Hypertension	31 (60.8%)
Previous stroke/TIA	21 (41.2%)
Major bleeding history	3 (5.9%)
LVEF, %	62.7 ± 3.3
eGFR < 60 ml/min/1.73 m^2^	2 (3.9%)
*Heart failure (NYHA)*	
I	4 (7.8%)
II	37 (72.6%)
III	10 (19.6%)
CHA2DS2-VASc score	2.9 ± 1.4
HAS-BLED score	2.1 ± 0.8
One-stop procedure	22 (43.1%)
*LAAC device type*	
WATCHMAN	37 (72.6%)
LAmbre	14 (27.4%)

Values are mean ± SD, or n(%). AF: atrial fibrillation; TIA: transient ischemic attack; LVEF: left ventricular ejection fraction; eGFR: estimated glomerular filtration rate; LAAC: left atrial appendage closure

The median follow-up time was 96 days (IQR 88–112) from LAAC to CCTA and 96 days (IQR 88–116) from LAAC to TEE. 86.3% (44/51) of patients underwent both CCTA and TEE on the same day, while the interval between CCTA and TEE follow-up for the remaining patients was less than 7 days. Follow-up TEE showed that 51.0% (26/51) of patients had PDL with a median width of 2.23 mm (IQR 1.80–2.41). 88.5% (23/26) of the leaks were mild (<3 mm) in size, while 11.5% (3/26) were moderate (3–5 mm). No leak exceeded 5mm. It is worth noting that PDL was observed in 12 patients after device implantation, and 75% (9/12) were found to have a persistent leak upon follow-up TEE. The contrast medium patency could be observed on CCTA images for all leaks detected using TEE.

**Fig. 2:**
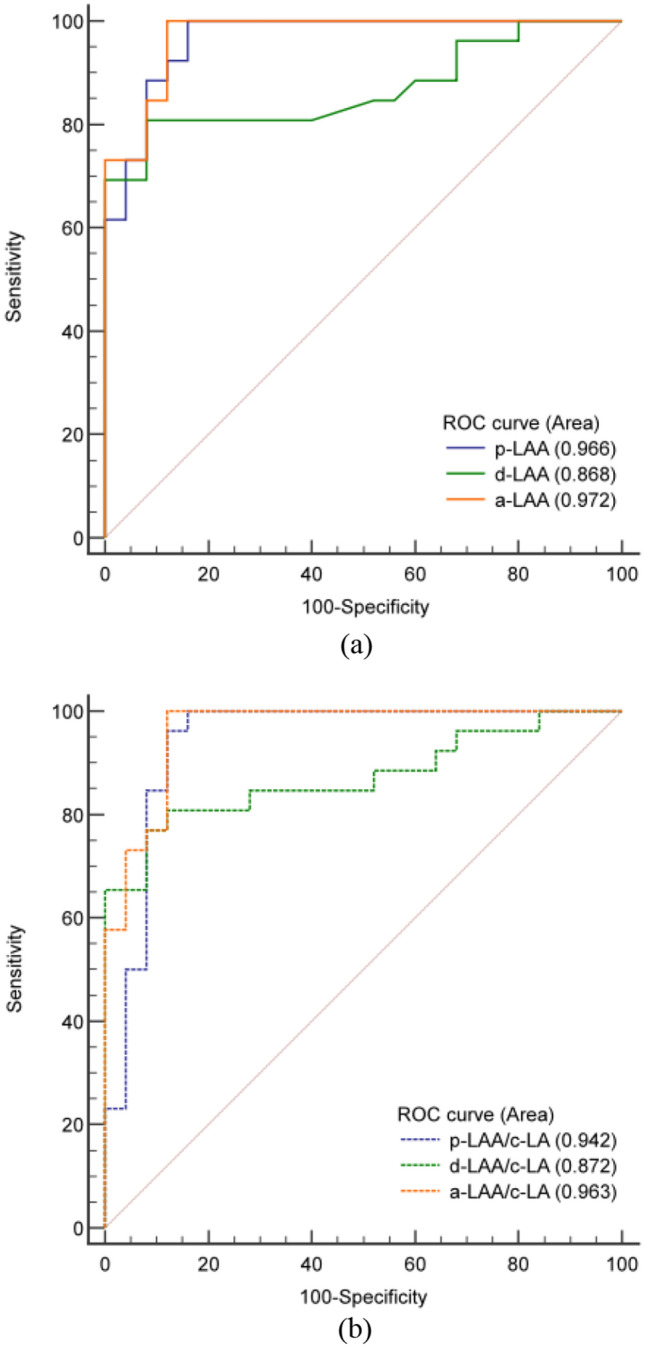
Receiver-operating characteristic (ROC) analysis in identifying PDL using TEE as the standard. **a** comparison of Hounsfield units of different regions in the left atrial appendage **b** comparison of the Hounsfield units ratios of different regions in the left atrial appendage to the center of the left atrium

The p-LAA and d-LAA of patients with LAA patency were 342.2 ± 96.7HU and 234.9 ± 135.8 HU (*p* < 0.05), while those with LAA occlusion had p-LAA of 88.9 ± 78.1HU and d-LAA of 54.6 ± 29.6HU (*p* < 0.05). The results of the ROC curve analysis and pairwise comparisons are shown in Table 2 and Fig. 2. The p-LAA had an AUC of 0.966 (95% confidence interval [CI] 0.873–0.997), d-LAA had an AUC of 0.868 (95% CI 0.743–0.946), and a-LAA had an AUC of 0.972 (95% CI 0.883–0.998). Pairwise comparisons showed a statistically significant difference in diagnostic performance between the d-LAA and a-LAA (*p* = 0.029). There were no significant differences between the a-LAA and p-LAA (*p* = 0.549) or between the d-LAA and p-LAA (*p* = 0.053). At the optimal cutoff for a-LAA of 115.5 HU, the sensitivity was 100%, the specificity was 88%, the PPV was 89.7%, the NPV was 100%, and the accuracy was 94.1%. At the optimal cutoff for p-LAA of 109 HU, the sensitivity was 100%, the specificity was 84%, the PPV was 86.7%, the NPV was 100%, and the accuracy was 92.2%.

**Table 2:** ROC analysis for the assessment of PDL using different measurement protocols with CCTA

	p-LAA	d-LAA	a-LAA	p-LAA/c-LA	d-LAA /c-LA	a-LAA/c-LA
AUC	0.966	0.868	0.972	0.942	0.872	0.963
SE	0.021	0.054	0.018	0.037	0.052	0.023
*p*-value*	< 0.001	< 0.001	< 0.001	< 0.001	< 0.001	< 0.001
95%CI of AUC	0.873–0.997	0.743–0.946	0.883–0.998	0.838–0.988	0.749–0.949	0.869–0.996
*Statistical results of diagnostic performance*						
Optimal cutoff value	109.0	75.0	115.5	0.459	0.219	0.305
Sensitivity, %	100.0	80.8	100.0	96.2	76.9	100.0
Specificity, %	84.0	92.0	88.0	88.0	92.0	88.0
PPV, %	86.7	91.3	89.7	89.3	90.9	89.7
NPV, %	100.0	82.1	100.0	95.7	79.3	100.0
Accuracy, %	92.2	86.3	94.1	92.2	84.3	94.1
*Statistical comparison of ROC curves, p-value*						
p-LAA	–	–	–			
d-LAA	0.053	–	–			
a-LAA	0.549	*0.029*	–			
p-LAA/c-LA	0.200	0.221	0.230	–	–	–
d-LAA/c-LA	0.053	0.764	*0.027*	0.226	–	–
a-LAA/c-LA	0.728	0.051	0.222	0.270	*0.047*	–

*p values show how AUC differs from 0.5. ROC: receiver-operating characteristic; PDL: peri-device leak; CCTA: cardiac computed tomography angiography; LAA: left atrial appendage; LA: left atrial; p/d/a-LAA: the HU measured in the proximal region and the distal region of the LAA, and the average of the two; c-LA: the HU measured in the center of the LA; AUC: area under the curve; SE: standard error; CI: confidence interval; PPV: positive predictive value; NPV: negative predictive value

The AUC was 0.942 (95% CI 0.838–0.988) for p-LAA/c-LA, 0.872 (95% CI 0.749–0.949) for d-LAA/c-LA, and 0.963 (95% CI 0.869–0.996) for a-LAA/c-LA. There were only marginally significant differences between d-LAA/c-LA and a-LAA/c-LA (*p* = 0.047). At the optimal cutoff for a-LAA/c-LA of 0.305, the sensitivity, specificity, PPV, NPV, and accuracy were the same as those mentioned above for a-LAA of 115.5 HU.

The left ventricular ejection fraction (LVEF) in patients without PDL (63.6 ± 2.5%) was greater than in those with PDL (61.8 ± 3.8%, *p* < 0.05). There were no significant differences in other parameters such as anatomical structure, device compression, or baseline characteristics (Table 3). Five patients without PDL on TEE were found to have p-LAA > 109.0 on the CCTA image. Two of these patients did not present significant leakage through the ostial peri-device gap, suggesting possible leakage from the fabric of the device [21, 22]. DRT occurred in only one patient with the WATCHMAN device. After switching from dabigatran to rivaroxaban and continuing anticoagulation for three months, the thrombus was significantly dissolved upon re-examination via TEE, shrinking from 23*7 mm to 14*4 mm. Follow-up CCTA, TEE, and re-examination TEE all revealed the presence of PDL measuring from 1 to 2 mm, with no significant change in size over 6 months.

**Table 3:** Comparison of baseline characteristics between patients with and without leaks using TEE

	PDL(*N* = 26)	No PDL (*N* = 25)	*p*-value
Sex, female	14 (53.8%)	9 (36.0%)	0.200
Age, years	64.9 ± 7.9	64.6 ± 6.9	0.893
Paroxysmal AF	7 (26.9%)	11 (44.0%)	0.202
CHA2DS2-VASc score	3.2 ± 1.5	2.6 ± 1.2	0.202
HAS-BLED score	2.2 ± 0.8	2.0 ± 0.7	0.260
*LAA shape*			
Cauliflower	15 (57.7%)	16 (64.0%)	0.823
Chicken-wing	10 (38.5%)	8 (32.0%)	
Cactus	1 (3.8%)	0 (0%)	
Windsock	0 (0%)	1 (4.0%)	
Maximum diameter of LAA orifice, mm	24.3 ± 4.5	22.3 ± 3.2	0.073
Maximum LAA depth, mm	22.0 ± 3.9	22.5 ± 2.8	0.607
LVEF, %	61.8 ± 3.8	63.6 ± 2.5	*0.034*
One-stop procedure	10 (38.5%)	12 (48.0%)	0.492
WATCHMAN	16 (61.5%)	21 (84.0%)	0.072
Device size, mm	29.3 ± 2.6	28.3 ± 3.4	0.421
Device compression rate, %	18.6 ± 3.9	20.4 ± 2.8	0.107
*Anticoagulation post LAAC*			
Rivaroxaban	22 (84.6%)	20 (80.0%)	0.848
Dabigatran	3 (11.5%)	4 (16.0%)	
Warfarin	1 (3.8%)	1 (4.0%)	

Values are mean ± SD, or n(%). AF: atrial fibrillation; LAA: left atrial appendage; LVEF: left ventricular ejection fraction; LAAC: left atrial appendage closure

## Discussion

The present study demonstrates that different ROI positions in CCTA affect the diagnostic performance of LAA HU values and LAA/LA HU ratio. We determined a-LAA and p-LAA to be the best parameters for identifying PDL, with cut-offs of 115.5 and 109.0, respectively. The diagnostic performance of the HU ratio and absolute LAA HU value was not statistically different. Our proposed measurement protocols provided a sensitivity of 76.9–100% and a specificity of 84–92% for PDL on CCTA images, using TEE as a standard. Therefore, CCTA can be a viable alternative to TEE for PDL evaluation post LAAC.

The identification of PDL via CCTA relies on determining the presence of a peri-device gap or contrast enhancement within the LAA. The majority of studies assess LAA patency using the HU of LAA or LAA/LA HU ratio in CCTA. As the criterion of ROI position in LAA have not been defined, consistency across patients is difficult to maintain. The ratio of LAA/LA for evaluating PDL in several studies ranges from 0.25 to 0.43 [12, 15, 16], which may be the result of different ROI selections in the LAA.

It is unclear whether the ROI should be positioned close to the orifice for maximal contrast agent concentration or at the mid or caudal end of the LAA to avoid metal interference when the PDL is present. To answer these questions, we proposed and compared different measurement protocols for PDL detection on CCTA images. Considering that the ROI in this study was set as a circle with a diameter of 1.6 mm, we divided the LAA into proximal and distal regions using 3.2 and 4.8 mm demarcation lines around the device. The 1.6 mm gap between the two regions was intended to create a clearer dividing line.

Using TEE as the gold standard, we found that there were indeed differences in the PDL diagnostic performance of HU measured in different regions. The diagnostic performance of HU in the distal region was inferior to that of the proximal region and the mean value. There was no statistical difference in AUC between the p-LAA and a-LAA. As a-LAA demonstrated greater AUC, specificity, PPV, and accuracy, we recommend a protocol that calculates the mean HU of the distal and proximal region. At the optimal cutoff for a-LAA of 115.5 HU, the sensitivity was 100%, the specificity was 88%, the PPV was 89.7%, the NPV was 100%, and the accuracy was 94.1%. In practice, calculating the mean value could increase the clinical workload. Therefore, p-LAA can also be used as an alternative protocol due to its convenience and intuitiveness. At the optimal cutoff for p-LAA of 109 HU, the sensitivity was 100%, the specificity was 84%, the PPV was 86.7%, the NPV was 100%, and the accuracy was 92.2%. In previous studies, the HU value in the LAA with a cutoff of 100 was identified as the best parameter for identifying LAA residual patency [12, 16]. The differences in threshold values may be influenced by ROI selection, but also by the confounding factors of individual differences such as heart rate rhythms, CCTA scan parameters, and image acquisition time which require more research to clarify.

The LAA/LA ratio did not demonstrate better diagnostic performance than HU attenuation in the LAA in this study. One possible cause of this is the confounding factors of individual differences mentioned above. Another is the lower LVEF in patients with PDL in this study. Low cardiac output is associated with low blood flow velocity, which can lead to delayed and altered contrast entry [23, 24]. Angelillis et al. [16] place the ROI similar to the proximal region established in our study. In that study, 0.43 was the best LAA/LA HU ratio for differentiating LAA patency. This is near the cutoff for p-LAA/c-LA of 0.459 in this study.

In our study, the LAA/LA HU ratio and LAA HU values in the proximal region were higher than in previous studies, probably due to the interference of metal instruments and increased local contrast agent concentration caused by blood flow pooling. Although the distal region was set up to avoid metal interference, during the data collection process, it was found that the blood flow of some patients was confined to the proximal end and did not spread to the distal end. This difference may have contributed to the deterioration of sensitivity in the distal region. However, it was observed that HU values and ratios in the distal region provided the best specificity and PPV, a fact which may be useful in identifying LAA patency in PDL patients with micro-contrast entry.

Gerhoff et al. [25] showed that the distribution of absolute HU values in LAA and the ratio of HU in LAA and LA differed significantly depending on the scanning phase. Therefore, using the absolute HU value in the proximal region of the LAA as the best and easiest method to detect PDL may not work with different scanners and scanning protocols. The LAA/LA ratio is more complex to calculate but may be more accurate when used at different centers.

The factors influencing the occurrence of PDL after LAA closure remain unclear. Our study showed that LVEF was lower in patients with occluded LAA compared to those with patent LAA, which is consistent with the previous study [26]. We did not find any other factors affecting PDL.

## Limitations

This study was carried out in a single center with a small sample, which may affect the validity of our results. Larger studies are needed to verify them. We did not further differentiate the intra-device or peri-device region. The clinical significance of PDL identified using CCTA is currently unclear. We did not use different scanners and scanning protocols to verify the generalizability of the results, and more research is needed to confirm this.

## Conclusions

CCTA can be used as a non-invasive examination after LAAC, especially for patients with contraindications to TEE. This study has shown that there are indeed differences in the PDL diagnostic performance of HU measured in different regions. The a-LAA > 115.5 is useful in identifying PDL. Due to its convenience and intuitiveness, p-LAA > 109.0 can also be used as an alternative protocol for a-LAA. The HU ratio of LAA/LA did not lead to improved diagnostic performance.
